# Comparison of the clinical and laboratory characteristics of neuromyelitis optica spectrum disorder with or without cerebrospinal fluid oligoclonal bands: a cohort with 36-month follow-up

**DOI:** 10.3389/fimmu.2025.1536853

**Published:** 2025-03-14

**Authors:** Wenbo Yang, Xiaoni Liu, Jie Wei, Hai Yu, Wanqing Wu, Jingguo Wang, Bo Deng, Xiaoqin Wu, Xiangjun Chen, Xiang Zhang

**Affiliations:** ^1^ Department of Neurology, Huashan Hospital, Fudan University and Institute of Neurology, Fudan University, National Center for Neurological Disorders, Shanghai, China; ^2^ Department of Neurology, NO. 905 Hospital of People's Liberation Army Navy affiliated to Naval Medical University, Shanghai, China; ^3^ Human Phenome Institute, Fudan University, Shanghai, China

**Keywords:** neuromyelitis optica spectrum disorder, oligoclonal bands, clinical characteristics, laboratory characteristics, 36-month follow-up

## Abstract

**Purpose:**

This study aimed to explore the significance of cerebrospinal fluid (CSF) oligoclonal bands (OCBs) in the clinical diagnosis and evaluation of neuromyelitis optica spectrum disorder (NMOSD).

**Methods:**

The demographic and clinical data of 143 aquaporin-4 immunoglobulin G (AQP4-IgG)-positive NMOSD patients were collected and analyzed, including the gender, age, clinical symptoms and signs, status of CSF OCBs, location and length of the affected spinal cord vertebral segments, Expanded Disability Status Scale (EDSS) at the first attack and at 36-month follow-up, relapse times within 36 months, concomitant connective tissue disease (CTD), and status of other autoimmune antibodies (oAIA).

**Results:**

There were 15 patients (10.5%) who were positive for OCBs (OCBs+). In contrast to those with negative OCBs (OCBs−), more OCBs+ cases had concomitant CTD [5/15 (33.3%) *vs*. 11/128 (8.6%), *p* = 0.014] and oAIA [9/15 (60.0%) *vs*. 37/128 (28.9%), *p* = 0.020]. OCBs+ patients had higher CSF cell counts [15.0 (27.0)/mm^3^ *vs*. 5.0 (12.0)/mm^3^, *p* = 0.008], higher IgG index [0.68 (0.23) *vs*. 0.52 (0.15), *p* < 0.001], and more relapses within 36 months [2.0 (3.0) *vs*. 1.0 (2.0), *p* = 0.039] than OCBs− patients. More OCBs+ patients had polynuclear cell predominance in the CSF than OCBs− patients (*p* = 0.032). There were no significant differences between the OCBs+ and the OCBs− patients in the distribution of lesion locations; the length of the affected spinal cord vertebral segments; the concentration of CSF protein and the albumin quotient; the EDSS score at the time of lumbar puncture and at 36-month follow-up, and the onset episode, the relapse, and cumulative clinical syndrome profiles (all *p* > 0.05).

**Conclusions:**

For AQP4-IgG-positive NMOSD patients, positivity for CSF OCBs is associated with higher CSF cell counts and a higher likelihood to have concomitant CTD and oAIA. OCBs+ is not uncommon in NMOSD and may predict more frequent relapses, but not a more serious illness.

## Introduction

The presence of oligoclonal bands (OCBs) in the cerebrospinal fluid (CSF) has long been identified as a key biomarker for multiple sclerosis (MS) and is now generally recognized as a substitute for dissemination in time and one of the diagnostic essentials for MS in the 2017 McDonald’s diagnostic criteria (1). However, OCBs can also be detected in several other neuroinflammatory diseases and central nervous system (CNS) infections, including Guillain–Barré syndrome, neuropsychiatric lupus erythematosus, CNS vasculitis, and neuromyelitis optica spectrum disorder (NMOSD), among others (2–6).

Neuromyelitis optica (NMO), also known as Devic’s disease, is a neuroinflammatory disease with optic neuritis (ON) and/or longitudinally extensive transverse myelitis (LETM), and it was once considered a subtype of MS. However, there is now a large body of evidence supporting that NMO is an independent disease entity presenting with more clinical manifestations except for ON and LETM, more like a spectrum of disease, and is nominated as NMOSD for its diverse manifestations (7–10). The anti-aquaporin-4 antibody (aquaporin-4 immunoglobulin G, AQP4-IgG) is considered a specific and an important diagnostic marker for NMOSD, which makes distinguishing NMOSD from MS easier (8–11).

Reports of the occurrence and the importance of OCBs in NMOSD are sparse, and the cohorts observed were small (12, 13). Do patients with NMOSD with or without OCBs have different clinical and/or laboratory features? Do the OCBs in NMOSD indicate higher relapse rates and/or more severe conditions? These questions remain to be elucidated. To further explore the significance of OCBs in the clinical diagnosis and evaluation of NMOSD, the relationship between OCB status and clinical and laboratory characteristics was analyzed in 143 AQP4-IgG-positive NMOSD patients in this study.

## Materials and methods

### Participants and data collection

**Figure 1:**
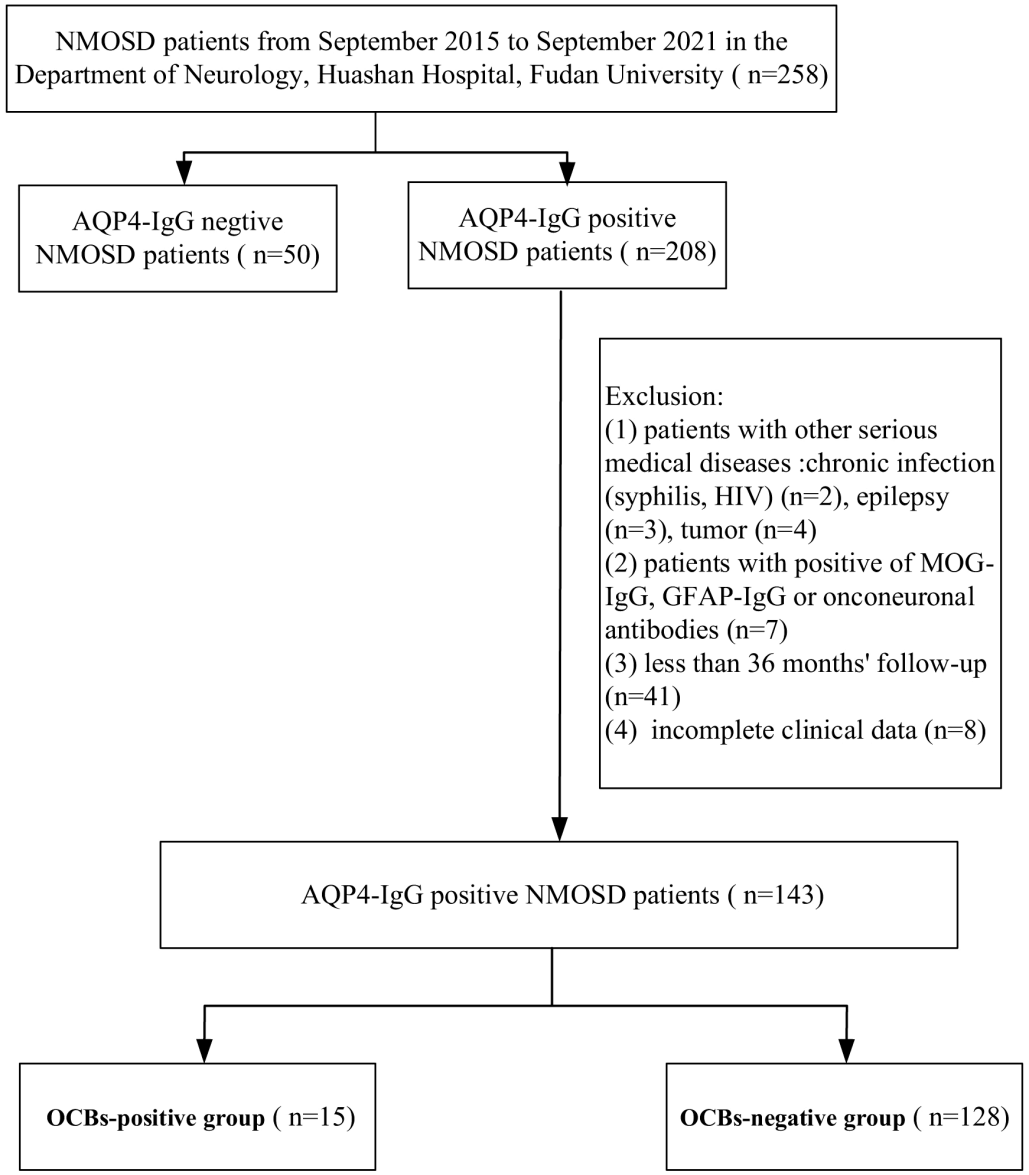
Flowchart of the aquaporin-4 immunoglobulin G (AQP4-IgG)-positive patients enrolled in this study. *NMOSD*, neuromyelitis optica spectrum disorder; *MOG-IgG*, anti-myelin oligodendrocyte glycoprotein immunoglobulin G; *GFAP-IgG*, anti-glial fibrillary acidic protein immunoglobulin G; *OCBs*, oligoclonal bands.

In this study, a total of 258 patients with NMOSD in the Department of Neurology, Huashan Hospital, Fudan University, from September 2015 to September 2021 were enrolled, from which a total of 143 AQP4-IgG-positive NMOSD patients were included (**Figure 1**). All patients met the 2015 International Panel for NMO diagnostic criteria (7). The clinical data of the NMOSD patients were collected, including the gender, age, clinical symptoms and signs, location and length of the affected spinal cord segments, status of OCBs, Expanded Disability Status Scale (EDSS) at the first attack and at the 36-month follow-up, relapse times within 36 months, concomitant connective tissue disease (CTD), and other autoimmune antibodies (oAIA).

The exclusion criteria were as follows: 1) patients with other serious medical diseases, chronic infection (e.g., syphilis, HIV, etc.), epilepsy, and tumors; 2) patients positive for anti-myelin oligodendrocyte glycoprotein immunoglobulin G (MOG-IgG), anti-glial fibrillary acidic protein immunoglobulin G (GFAP-IgG), or onconeuronal antibodies; 3) less than 36 months of follow-up; and 4) incomplete clinical data.

Fresh paired serum and CSF samples were obtained within 30 days from the onset of the recent episode. Samples were stored at 2–8°C and analyzed within 15 days. This study was approved by the Ethics Committee of Huashan Hospital, Fudan University.

All patients were enrolled at the time of lumbar puncture and had received corticosteroid therapy. During 36 months of follow-up, 60 patients received azathioprine, 62 received mycophenolate mofetil, 14 received rituximab, and 7 received other medications (e.g., tacrolimus, cyclophosphamide, methotrexate, and cyclosporine) (**Table 1**).

**Table 1:** Demographic and clinical characteristics of neuromyelitis optica spectrum disorder (NMOSD) patients with aquaporin-4 immunoglobulin G (AQP4-IgG) categorized based on the presence or absence of oligoclonal bands (OCBs).

	OCB-positive	OCB-negative	*Z* value	*p*-value
No. of cases, *n*	15	128		
Men, *n* (%)	2 (13.33%)	12 (10.17%)		*0.643*
Age (years), median (IQR)	43 (22)	45.5 (18)	−0.547	0.584
EDSS at enrollment, median (IQR)	4.0 (1.0)	4.0 (2.0)	−0.773	0.439
EDSS at 3-year follow-up, median (IQR)	3.0 (2.0)	3.0 (2.4)	−0.255	*0.799*
Relapse time within 36 months of follow-up, median (IQR)	2.0 (3.0)	1.0 (2.0)	−2.064	***0.039***
CSF protein (mg/L), median (IQR)	571.0 (381.0)	467.0 (286.0)	−1.815	*0.070*
CSF white cell counts (/mm^3^), median (IQR)	15.0 (27.0)	5.0 (12.0)	−2.635	***0.008***
IgG index, median (IQR)	0.68 (0.23)	0.52 (0.15)	−3.959	***0.000***
Concomitant CTD, *n* (%)	5 (33.3%)	11 (8.6%)		***0.014***
Concomitant other autoantibodies, *n* ( %)	9 (60.0%)	37 (28.9%)		***0.020***
Imaging lesions evidenced by MRI at enrollment, *n* (%)				
Involving the brain and brainstem	8 (53.3%)	48 (37.5%)		*0.235*
Involving the optic nerve	9 (60.0%)	69 (53.9%)		*0.654*
Involving the spinal cord	14 (93.3%)	99 (77.3%)		*0.195*
Lesion length in the spinal cord, median (IQR)	6.0 (7.0)	4.0 (4.0)	−1.173	*0.241*
DMTs, *n* (%)				*0.944*
AZA	6 (40.0%)	54 (42.2%)		
MMF	6 (40.0%)	56 (43.8%)		
RTX	2 (13.3%)	12 (9.4%)		
Other	1 (6.7%)	6 (4.7%)		

“Other” includes tacrolimus, cyclophosphamide, methotrexate, and cyclosporine.

EDSS, Expanded Disability Status Scale; CSF, cerebrospinal fluid; CTD, connective tissue disease; IQR, interquartile range; DMTs, disease-modifying therapies; AZA, azathioprine; MMF, mycophenolate mofetil; RTX, rituximab.

P-values are uniformly emphasized in italics and p-values less than 0.05 are emphasized in bold italics.

The serum AQP4-IgG, MOG-IgG, and GFAP-IgG concentrations of all enrolled patients were determined with a cell-based assay (CBA) using the kit from Simcere Medical Diagnosis Co., Ltd. (Nanjing, China). Antibody titers ≥1:10 were considered as positive. Other autoimmune antibodies [i.e., antinuclear antibody (ANA), anti-Sjogren’s syndrome type A (SSA) and type B (SSB) antibodies, anti-double-stranded DNA (ds-DNA) antibody, etc.] were detected using the diagnostic kit from EUROIMMUN Medical Diagnostics (Beijing, China). To avoid inter-assay variations, the same positive control, negative control, and blank control were used as the reference samples for each detection.

### Evaluation of the blood–CSF barrier and intrathecal IgG synthesis

The IgG and albumin concentrations in the serum and CSF were determined using the turbidimetric scattering method. The blood–CSF barrier (BCB) function was assessed using the CSF/serum albumin quotient: (*Q*_Alb_) = CSF − Alb (mg/L)/serum − Alb (g/L), with BCB dysfunction defined as *Q*_Alb_ > 4 + (age/15) (5, 14).

The IgG index, calculated as (CSF IgG/serum IgG)/(CSF albumin/serum albumin), was a measure of the intrathecal IgG synthesis adjusted for the degree of damage of the BCB. A value below 0.7 was considered normal (14).

### OCB detection

The detection of OCBs was performed using a Sebia HYDRASYS 2 Isofocusing system (PN1211, Lisses, France) according to the manufacturer’s instructions. Briefly, the CSF and serum samples were run in parallel on an agarose gel. After electrophoresis, the gel was incubated with peroxidase-labeled anti-IgG antibodies, and the bands were displayed using TTF1/2 chromogenic agents (15). The two inspectors interpreted the electrophoresis results independently according to the following interpretations: type I, no bands in both the serum and CSF; type II, more than two bands in the CSF, but no band in the serum; type III, additional bands in the CSF despite bands in the serum; type IV, symmetrically separated OCB bands in the serum and CSF; and type V, twin bands with regular and periodic spacing in both the serum and CSF (16, 17). Types II and III were considered positive for CSF OCBs (OCBs+), while the remaining three types were considered negative (OCBs−).

### Statistical analysis

SPSS 19.0 software was used for statistical analysis. The normality of continuous data was assessed using the Shapiro–Wilk test. Data with normal distribution are expressed as $\bar{\chi }$ ± SD, while those with non-normal distribution are expressed as median (interquartile range, IQR). Comparison of the classification variables between groups was performed using Pearson’s *χ* ^2^ test or Fisher’s exact probability method or the Cochran–Mantel–Haenszel test. Pairwise comparisons were performed using an independent two-sample *t*-test or the Mann–Whitney *U*-test, as applicable. A *p* < 0.05 was considered statistically significant.

## Results

### Demographic data and clinical manifestations at enrollment

A total of 143 patients with NMOSD, including 14 men and 129 women (men/women = 1:9.2), were enrolled in this study (**Figure 1**). The median age at enrollment was 44 (18) years. In the OCBs+ group, 15 patients were included, two men and 13 women (men/women = 1:6.5), with a median age of 43 (22) years. The OCBs− group comprised 128 patients, 12 men and 116 women (men/women = 1:9.7), with a median age of 46 (18) years. There were no significant differences in the gender and age distribution between the two groups (*p* = 0.643 for gender and *p* = 0.685 for age).

Of the 143 patients, there were 16 (11.2%) patients with CTD and 46 (32.2%) patients with oAIA.

The median relapse time within 36 months was 2.0 (2.0), and the EDSS at baseline and at 36 months were 4.0 (2.0) and 3.0 (2.0), respectively. With regard to the lesion distribution revealed by MRI, of the 143 cases, 56 (39.2%) had intracranial lesions, 78 (54.5%) had optic nerve lesions, and 113 (79.0%) had spinal cord (SC) lesions with the median length of 4.0 (4.0) vertebral segments.

Considering the possible impact of drugs on the laboratory indicators, the differences in drug use among patients were compared with the different OCB status. In the OCBs+ group, six received azathioprine, six received mycophenolate mofetil, two received rituximab, and one received other medications (tacrolimus). In the OCBs− group, 54 received azathioprine, 56 received mycophenolate mofetil, 12 received rituximab, and 6 received other medications (e.g., tacrolimus, cyclophosphamide, methotrexate, and cyclosporine). There were no differences in the therapies between patients with positive and negative OCBs (*p* = 0.944) (**Table 1**).

### Comparison of the CSF parameters between the OCBs+ and OCBs− NMOSD patients with AQP4-IgG

In general, the median CSF cell count and protein level were 5 (15)/mm^3^ and 487 (284) mg/L, respectively. Among the 143 cases, 49 (34.3%) had BCB destruction and 20 (14.0%) had an elevated IgG index. Moreover, there were 128 (89.5%) patients with negative OCBs and 15 (10.5%) patients with positive OCBs, including 11 type II and 4 type III.

For CSF biochemistry, OCBs+ patients had higher white blood cell counts than OCBs− patients [15.0 (27.0) *vs*. 5.0 (12.0)/mm^3^, *p* = 0.008]. Detailed white cell differentiation analysis of the 143 patients revealed that 130 (90.9%) exhibited a predominance of mononuclear cells in their CSF [11/15 (73.3%) in the OCBs+ group and 119/128 (93.0%) in the OCBs− group], while 13 (9.1%) showed a predominance of polynuclear cells [4/15 (26.7%) in the OCBs+ group and 9/128 (7.0%) in the OCBs− group]. Notably, the proportion of patients with a predominance of polynuclear cells in the CSF was significantly higher in the OCBs+ group compared with the OCBs− group (4/15 *vs*. 9/128, *p* = 0.032). There were no statistically significant differences for the CSF protein levels [571.0 (381.0) mg/L *vs*. 467.0 (286.0) mg/L, *p* = 0.070] between these two groups.

In the OCBs+ group, 8 out of 15 (53.3%) patients had elevated *Q*_Alb_ with a median IgG index of 0.68 (0.23), whereas in the OCBs− group, 41 out of 128 (32.0%) cases had elevated *Q*_Alb_ with a median IgG index of 0.52 (0.15). OCBs+ patients had a higher IgG index than OCBs− patients (*p* < 0.001) (**Table 1**).

### Comparison of concomitant CTD and oAIA between the OCBs+ and OCBs− NMOSD patients

In contrast to OCBs− patients, more OCBs+ patients had concomitant CTD [5/15 (33.3%) *vs*. 11/128 (8.6%), *p* = 0.014] and oAIA [9/15 (60.0%) *vs*. 37/128 (28.9%), *p* = 0.020] (**Table 1**).

### Comparison of involved CNS lesions revealed by MRI between the OCBs+ and OCBs− patients with NMOSD at enrollment

During the baseline MRI evaluation at enrollment, brain/brainstem involvement was detected in 8 out of 15 (53.3%) OCB+ patients compared with 48 out of 128 (37.5%) in the OCB− group. Optic nerve lesions were identified in 9 out of 15 (60.0%) and in 69 out of 128 (53.9%) patients. SC abnormalities comprised the most prevalent imaging feature, which were present in 14 out of 15 (93.3%) OCB+ cases and in 99 out of 128 (77.3%) OCB− cases. No statistically significant intergroup differences were observed in the lesion distribution patterns (all *p* > 0.05) (**Table 1**).

### Comparison of the clinical manifestations between the OCBs+ and OCBs− patients with NMOSD

At the time of enrollment, myelitis was present in 14 out of 15 (93.3%) OCB+ patients and in 99 out of 128 (77.3%) OCB− patients, with median SC lesion lengths of 6.0 (7.0) and 4.0 (4.0) vertebral segments, respectively. No statistically significant intergroup differences were identified in either myelitis prevalence or lesion length (both *p* > 0.05) (**Table 1**).

For OCBs+ patients, the median EDSS at enrollment was 4.0 (1.0), while it was 3.0 (2.0) at the 36-month follow-up; the median relapse times within 36 months was 2.0 (3.0). For OCBs− patients, the median EDSS was 4.0 (2.0) at lumbar puncture and was 3.0 (2.5) at the 36-month follow-up, and the median relapse time at the 36-month follow-up was 1.0 (2.0). OCBs+ cases had more relapses than OCBs− patients (*p* = 0.039). However, there were no significant statistical differences in the EDSS between the two groups, either at enrollment or at the 36-month follow-up (both *p* > 0.05) (**Table 1**).

Among the 15 OCBs+ patients, the onset episode manifested as myelitis in 10 (66.7%) cases, ON in 8 (53.3%), and area postrema syndrome in 2 (13.3%). Among the 128 OCBs− patients, the onset episode included myelitis in 83 (64.8%) cases, ON in 54 (42.2%), and area postrema syndrome in 16 (12.5%). No statistically significant differences were observed between these two groups (all *p* > 0.05) (**Table 2**).

**Table 2:** Clinical syndromes of neuromyelitis optica spectrum disorder (NMOSD) patients based on the presence or absence of oligoclonal bands (OCBs).

	OCB-positive	OCB-negative	*p*-value
Onset syndrome			
Myelitis	10/15 (66.7%)	83/128 (64.8%)	*0.842*
Optic neuritis Area postrema syndrome	8/15 (53.3%) 2/15 (13.3%)	54/128 (42.2%) 16/128 (12.5%)	*0.410* *>0.999*
Relapses during the 36-month follow-up			
Myelitis	10/11 (90.9%)	51/66 (77.3%)	*0.442*
Optic neuritis Area postrema syndrome	3/11 (27.3%) 0/11 (0.0%)	30/66 (45.5%) 1/66 (1.5%)	*0.335* *>0.999*
Cumulative clinical involvement after the 36-month follow-up			
Myelitis	14/15 (93.3%)	107/128 (83.6%)	*0.468*
Optic neuritis Area postrema syndrome	9/15 (60.0%) 2/15 (13.3%)	68/128 (53.1%) 17/128 (13.3%)	*0.613* *>0.999*

P-values are uniformly emphasized in italics.

During the 36-month follow-up period, disease relapses occurred in 11 OCBs+ patients, manifesting as myelitis in 10 (90.9%) cases, ON in 3 (27.3%), and area postrema syndrome in 0 (0%). Among the OCBs− patients, 66 experienced relapses, presenting as myelitis in 51 (77.3%), ON in 30 (45.5%), and area postrema syndrome in 1 (1.5%). Again, no statistically significant differences were observed (all *p* > 0.05) (**Table 2**).

At the 36-month follow-up, the cumulative clinical involvement among all patients was as follows: in the 15 OCBs+ patients, myelitis was observed in 14 (93.3%) cases, ON in 9 (60%), and area postrema syndrome in 2 (13.3%). In the 128 OCBs− patients, myelitis was observed in 107 (83.6%) cases, ON in 68 (53.1%), and area postrema syndrome in 17 (13.3%). The observed variations demonstrated no statistically significant differences between the two groups (all *p* > 0.05) (**Table 2**).

## Discussion

OCBs are well known for their important diagnostic value and high prevalence in MS (1, 2, 18–20). However, CSF OCBs are not a specific indicator for MS and can also be identified in other diseases, particularly neuroinflammatory diseases (6, 17). In this study, the results showed that 10.5% of NMOSD patients with AQP4-IgG were positive for CSF OCBs. These results were consistent with a few previous studies indicating that NMOSD cases may also have intrathecal synthesis, but the positivity for OCBs was much lower than that in MS (12, 13, 20, 21). It also suggested that caution should be taken when making a diagnosis between MS and NMOSD, and one should carefully integrate the history and the clinical symptoms and signs together with CSF analysis, neuroimaging, and serological testing of AQP4-IgG, MOG-IgG, and oAIA before drawing a conclusion. CSF OCBs do not simply equate to a diagnosis of MS.

This study showed that OCBs+ NMOSD patients had higher CSF cell counts and a higher percentage of concomitant CTD compared with OCBs− NMOSD patients. It is known that a few patients diagnosed as CTD may have CNS symptoms and abnormal CSF examination (2, 22–24), which may be one of the reasons for the discrepancy in the CSF cell counts. As the cell counts are not abnormal in most MS cases, it is extremely noteworthy to pay attention to the higher CSF cell counts in the differentiation of NMOSD from MS.

In addition, while mononuclear cells predominated in the CSF of NMOSD patients overall, a significantly higher prevalence of multinuclear cell predominance was identified in OCBs+ patients compared with their OCBs− counterparts. Given that the cytological analysis of CSF in this study did not further differentiate the cell types into lymphocytes, monocytes, neutrophils, eosinophils, or basophils, the clinical significance of this finding remains uncertain. We propose further investigating this observation in future studies by increasing the sample size and conducting more detailed cytological analyses of the CSF.

Appropriate disease-modifying therapies (DMTs) can reduce relapses and control disease progression. Therefore, when comparing the frequency of relapses and the degree of disability caused by the disease in different patients during follow-up, it is necessary to consider the DMTs as a confounding factor. No significant differences were found when we compared the therapies between patients with different OCB status. OCBs have been reported to be an important risk factor for the conversion of clinically isolated syndromes to clinically definite MS (25, 26) and to be an indicator of more frequent recurrences and a more severe disease (27–30). In this study, there were no significant statistical differences in the EDSS between the OCBs+ and OCBs− patients at the time of enrollment or at the 36-month follow-up. However, there was a significant difference in the relapse times within 3 years. These results suggest that OCBs+ in NMOSD may represent a higher relapse rate, but not necessarily a more severe disease when properly treated.

There have been many reports that OCBs+ can persist for a long time in MS, with steroids and even most DMTs not being able to turn these negative (18, 31). There are only a few studies on the longitudinal observation of OCBs in patients with NMOSD, and the results are inconsistent. Bergamaschi et al. reported that all NMOSD patients with OCBs+ (three cases) turned negative within the following 17 months (13). However, as observed by Nakamura et al., one patient with NMOSD had OCBs+ at 22 years after onset, but the OCBs on his repeated CSF examination were still positive, with the same banding pattern even at 25 years after onset (12). As repeated lumbar puncture (LP) is invasive and unacceptable for many patients, as well as unnecessary for the follow-up of NMOSD, in this study, only six patients were tested twice for CSF-OCBs, of whom two patients had OCBs that changed from positive to negative and one OCBs+ and three OCBs− patients had an unchanged OCB status over 36 months. Thus, the status of OCBs may be variable in NMOSD; however, at what stage of the disease does this change occur remains an unanswered question. Further research is needed to consider the variability of OCBs as a marker for NMOSD and MS.

In fact, our original experimental design included patients with NMOSD who met the 2015 International Panel for NMO diagnostic criteria, regardless of whether they were positive for AQP4-IgG or not. However, after discussion with some reviewers, considering that AQP4-IgG-negative NMOSD patients have a higher disease heterogeneity and may have more uncertain factors (such as the low detection efficiency of AQP4-IgG and the presence of other possible pathogenic antibodies), we narrowed the scope of enrolled patients to AQP4-IgG-positive NMOSD patients in the study. This study has several limitations. It is a single-center study, and the number of enrolled patients with OCBs+ was not relatively large. In addition, a few patients with NMOSD were followed up with repeated CSF examinations to observe long-term changes in the OCB status. Therefore, multicenter prospective studies are needed in the future.

In summary, for AQP4-IgG-positive NMOSD patients, positive CSF OCBs are associated with higher CSF cell counts and a higher likelihood of concomitant CTD and oAIA. OCBs+ is not uncommon in NMOSD and may predict more frequent relapses, but not a more serious illness.

## Data Availability

The raw data supporting the conclusions of this article will be made available by the authors, without undue reservation.
